# Physiology-Informed Blink Artefact Detection from Bilateral Frontal EEG Under Asymmetric Interference

**DOI:** 10.3390/bioengineering13101208

**Published:** 2026-10-16

**Authors:** Weiqiang Zhang, Menglong Li, Yujie Zhang, Jingyi Cao, Feng Shen, Xiaohui Liu, Chen Chen, Yang Li

**Affiliations:** 1 College of Biomedical Engineering, Fudan University, Shanghai 200433, China; zhangwq@fudan.edu.cn; 2 Human Phenome Institute, Fudan University, Shanghai 201203, Chinayujie_z1008@163.com (Y.Z.);; 3 Department of Cardiovascular Surgery, Shanghai General Hospital, Shanghai Jiao Tong University School of Medicine, Shanghai 200080, China; drjingyicao@163.com (J.C.); sf02048207@163.com (F.S.); 4 Micro-Nano System Centre, School of Information Science and Technology, Fudan University, Shanghai 200433, China; liuxiaohui21@m.fudan.edu.cn; 5 Center for Medical Research and Innovation, Shanghai Pudong Hospital, Fudan University Pudong Medical Center, Shanghai 201399, China

**Keywords:** electroencephalography, blink detection, bilateral verification, ocular artefacts, signal processing

## Abstract

Eye blinks contaminate frontal electroencephalography (EEG), while asymmetric disturbances can cause false detections. We present a physiology-informed detector that combines waveform correlation, peak-time agreement, amplitude similarity, and symmetric-component dominance in an explicit event-verification rule. It uses two frontal channels without supervised model fitting. Evaluation on raw ZuCo 1.0 recordings included 260 eligible blocks from 12 participants, totalling 1291.61 min and 8611 eye-tracker reference intervals. Scalp and periocular pairs were evaluated separately. On the scalp pair, participant-mean precision was 0.731, recall 0.774, F1 0.734, and false positives 1.469 per minute. At fixed settings, false-positive rates were lower than with single-channel thresholding and bilateral averaging (both Holm-adjusted *p* = 0.005859), and remained lower than those of four rule-based controls across five perturbation conditions. Threshold-selected simple controls achieved higher precision at similar recall. Component ablations quantified the effects of the verification checks on sensitivity and false positives. The offline Python detector required a median of 2.13 ms per minute of recorded data on an Apple M1 Pro. These results establish a computationally inexpensive approach that makes blink-event decisions directly inspectable through bilateral signal measurements.

## 1. Introduction

Electroencephalography (EEG) is a widely used non-invasive technique for measuring brain activity in clinical and cognitive neuroscience research [1]. Eye blinks produce large potentials at frontal electrodes and can obscure neural activity or bias subsequent analysis [2,3]. Reliable blink detection is therefore an important part of EEG processing [4]. This need extends to wearable recordings, where movement and unequal electrode contact can introduce additional disturbances [5,6,7]. Reading studies illustrate the different roles of these signals: beta-band EEG has been used to estimate reading comprehension, while camera-derived blink measures have been combined with vocal features to study mind wandering [8,9]. Joint analysis of blink features and prefrontal EEG has also been used for driver fatigue detection [10]. Detecting a blink artefact in EEG and using blinking as a behavioural measure are related but distinct tasks.

Existing blink detectors use amplitude thresholds, waveform morphology, source separation, or learned features. Egambaram et al. combined Fp1–Fp2 correlation with amplitude-based thresholding, and Cao et al. incorporated frontal-channel correlation and amplitude differences into a Gaussian-mixture approach. BLINKER selects events through waveform fits and quality criteria, whereas Blink builds a recording-specific waveform profile. These approaches establish the roles of bilateral information and waveform structure in blink detection [11,12,13,14].

Recent work extends blink detection to compact acquisition systems and short processing windows. RT-Blink combines boundary detection over short sliding windows with a trained random forest classifier for a single frontal channel [15]. VME-DWT locates blink intervals by variational mode extraction and applies wavelet filtering within those intervals [16]. A portable two-channel forehead platform has also been used to classify blinks and eye movements from extracted EEG features [17].

Source-separation and learning methods offer additional ways to distinguish ocular from neural activity [18,19,20,21]. Convolutional variational autoencoders have also been used to identify blink-related latent components in EEG topographic maps [22]. Their suitability depends on the available channels, the training data, and the intended application; compact machine-learning models are also possible [23,24]. Simple rules remain useful when decisions must be easy to inspect. Their central difficulty is that a large transient in a frontal channel need not be a blink.

High correlation can persist even when the two channels differ markedly in amplitude. We therefore combine waveform correlation, peak-time agreement, amplitude similarity, and symmetric-component dominance in a physiology-informed blink detector. A sum/difference decomposition supplies the shared and unequal signal components. Each candidate is then accepted or rejected by an explicit rule linking the decision to these bilateral measurements. The resulting detector uses two input channels, requires no supervised model fitting, and makes event-level decisions directly inspectable.

We evaluate scalp and periocular pairs separately against eye-tracker reference events and report participant-level uncertainty. Comparisons with direct rule-based controls, BLINKER, and a supervised candidate classifier quantify detection accuracy and computational cost. Component ablations, participant-held-out threshold selection, and controlled perturbations establish how the verification criteria and operating settings affect false positives and sensitivity.

## 2. Materials and Methods

### 2.1. Physiological Basis

The eye has a standing corneo-retinal potential, with the cornea positive relative to the posterior eye. Eyelid movement and changes in the conductive path around the eye contribute to the frontal blink waveform. Spontaneous blinking involves coordinated bilateral eyelid movement, which can produce similar temporal waveforms at homologous frontal sites. Recorded amplitudes also depend on anatomy, reference choice, and electrode contact. These properties motivate joint assessment of waveform similarity, timing, and relative amplitude [2,25,26,27,28].

Bilateral consistency provides a physiological constraint for event verification. A candidate with similar waveforms and timing across the two sites satisfies this constraint, whereas an unequal disturbance can violate it. We express this relationship through measurable acceptance criteria.

### 2.2. Proposed Detector

**Figure 1:**
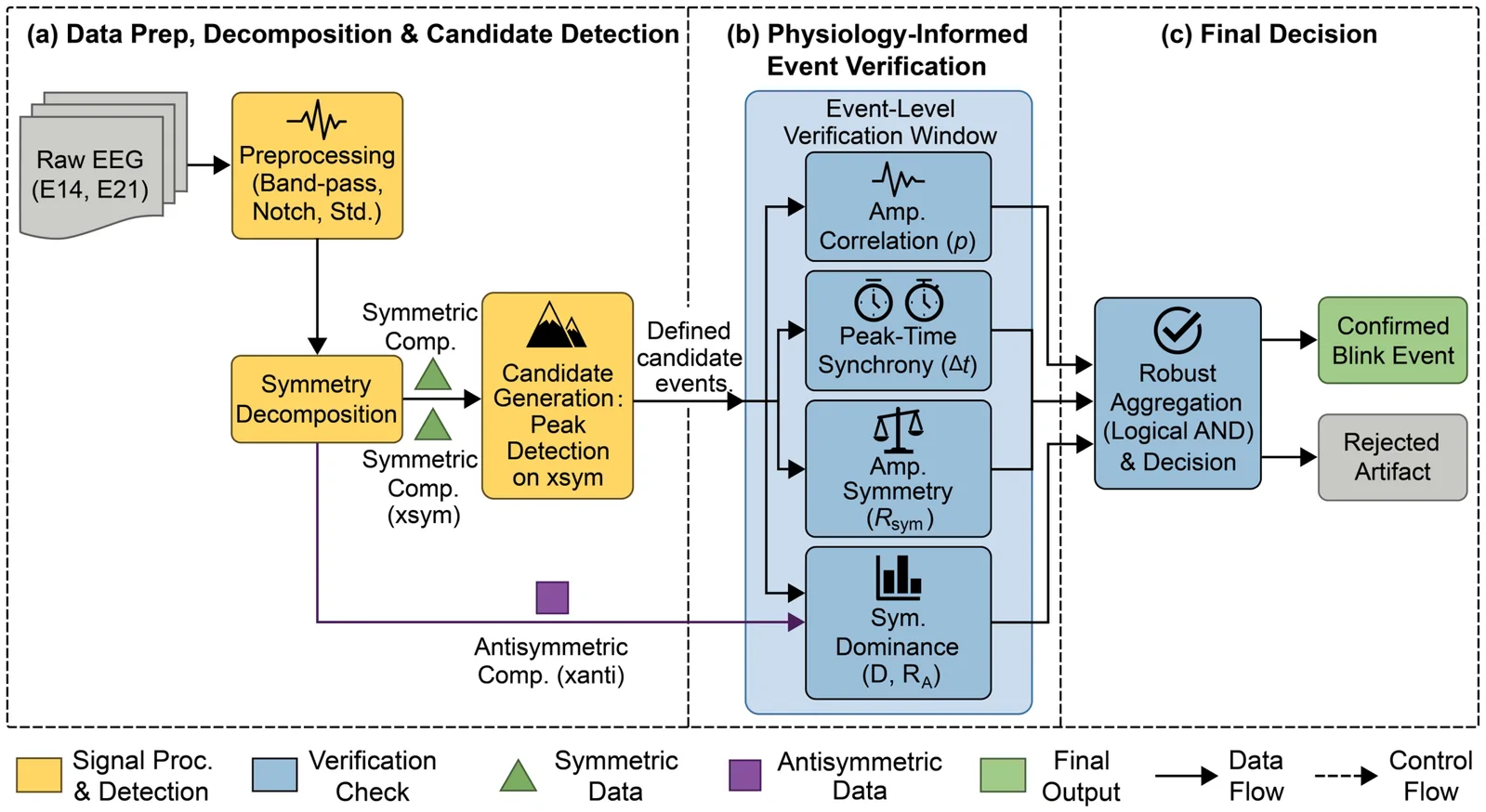
Candidate generation and bilateral verification for E14/E21; the same pipeline applies to E22/E9. Absolute symmetric peaks pass correlation, timing, amplitude-symmetry, and dominance checks (*D* and $R_{A}$) under logical AND. Section 2.2 gives duration, slope, and merging rules; eye-tracker intervals are evaluation-only.

Figure 1 summarizes candidate generation and bilateral verification.

#### 2.2.1. Symmetric and Antisymmetric Components

For standardized left and right signals $x_{L}\left(t\right)$ and $x_{R}\left(t\right)$, define

(1) $$x_{S}\left(t\right)=\frac{x_{L}\left(t\right)+x_{R}\left(t\right)}{2},\;x_{A}\left(t\right)=\frac{x_{L}\left(t\right)-x_{R}\left(t\right)}{2}.$$

This standard common/differential-mode transform separates shared and unequal activity for the subsequent event-verification tests.

#### 2.2.2. Candidate Detection

With $u\left(t\right)=|x_{S}\left(t\right)|$, the block-level threshold is

(2) $$h=\max\{\mu \left(u\right)+3.8\sigma \left(u\right),\;Q_{0.95}\left(u\right)\},$$

where $Q_{p}$ denotes a quantile. SciPy’s local-maximum detector finds peaks of *u* above *h*, separated by at least 12 samples at 500 Hz. Around each peak, the boundary search proceeds in both directions for at most 350 ms and stops at the first sample below 0.3h. If a crossing is absent on one side, that boundary remains at the peak, as in the supplied reference implementation. Candidate intervals shorter than 50 ms or longer than 700 ms are rejected.

Candidate spacing limits duplicate peak candidates, while event merging consolidates nearby accepted intervals. The respective settings are 12 samples and 200 ms. Both signal polarities can produce candidates because detection uses absolute amplitude; the subsequent bilateral checks determine event acceptance.

#### 2.2.3. Bilateral Verification

Within a candidate window *W*, correlation is calculated after smoothing each channel with a 20 ms moving average. We denote the smoothed signals by ${\bar{x}}_{L}$ and ${\bar{x}}_{R}$ and the sampling rate by $f_{s}$ (500 Hz). The four verification groups are

(3) $$\rho =\mathrm{corr}({\bar{x}}_{L},{\bar{x}}_{R})\geq 0.82,$$

(4) $$\Delta t=|\underset{W}{\mathrm{arg max}}|x_{L}|-\underset{W}{\mathrm{arg max}}|x_{R}\left|\right|/f_{s}\leq 80\;\mathrm{ms},$$

(5) $$R_{\mathrm{sym}}=\frac{|A_{L}-A_{R}|}{\max(A_{L},A_{R})}\leq 0.32,\;A_{j}=Q_{0.95}\left(\right|{\bar{x}}_{j}\left|\right),$$

(6) $$D=\frac{{\mathrm{median}}_{W}\left|x_{S}\right|}{{\mathrm{median}}_{W}\left|x_{A}\right|+10^{-10}}\geq 1.3,\;R_{A}=\frac{Q_{0.95}\left(\right|x_{A}\left|\right)}{Q_{0.95}\left(\right|x_{S}\left|\right)}\leq 0.7.$$

The fourth group comprises both *D* and $R_{A}$. Undefined correlation or zero channel amplitude leads to rejection. In addition, the largest absolute first difference within the candidate must be at least $1.5\sigma (\Delta x_{S})$, where the standard deviation is computed over the block. All criteria are combined using logical AND to produce a deterministic acceptance decision.

Accepted events are ordered by onset and merged when the gap from the preceding event’s end to the next event’s start is less than 200 ms. The merged event spans the union of the intervals and retains the larger peak. The 200 ms setting is an empirical event-consolidation rule. We assess its influence at gaps of 100, 150, 250, and 300 ms with all other parameters fixed.

Table 1 and the accompanying parameter file specify the detector settings. These values were developed using ZuCo and held fixed throughout the present evaluation. All eligible recordings are evaluated with the same configuration. Section 2.5 separately describes threshold selection for the controls, with each test participant excluded from that selection.

Algorithm 1 summarizes candidate generation, verification, and event merging.
**Algorithm 1** Bilateral verification with explicit candidate and merge rules**Input:** Preprocessed $x_{L},x_{R}$, sampling rate $f_{s}$, parameters in Table 1
  1:Compute $x_{S},x_{A},u=\left|x_{S}\right|$ and the block-level threshold *h*  2:Find local maxima above *h*, with the minimum candidate spacing  3:**for** each candidate peak **do**  4:    Search for 0.3h crossings within ±350 ms  5:    Reject if duration is outside 50–700 ms  6:    Calculate correlation, peak lag, amplitude asymmetry, and dominance  7:    Accept only if all four verification groups and the slope check pass  8:**end for**  9:Sort accepted intervals and merge adjacent intervals with gap <200 ms**Output:** Accepted blink intervals and peak locations


### 2.3. Dataset, Channels, and Reference Events

#### 2.3.1. Raw Data and Channel Provenance

**Figure 2:**
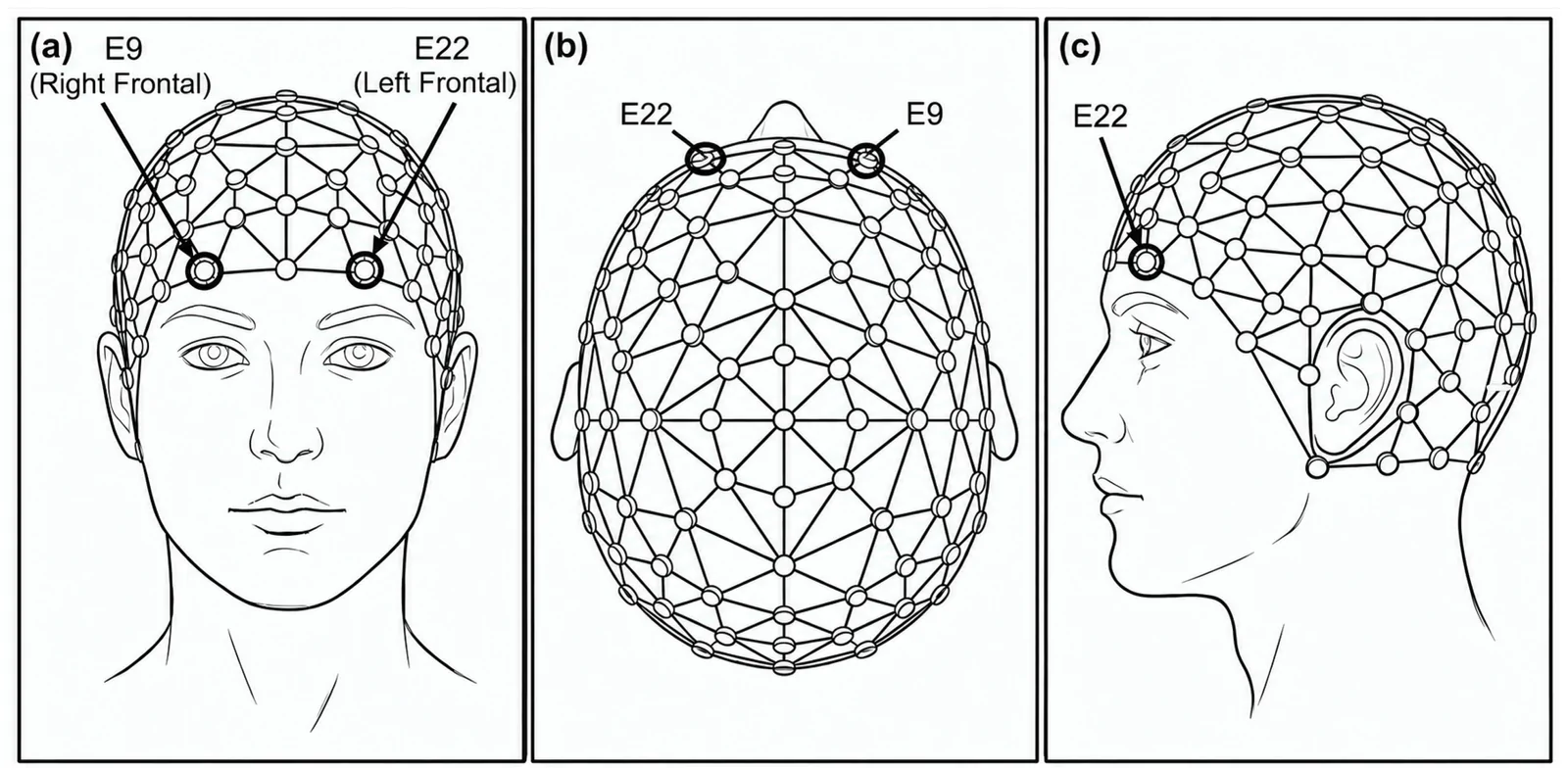
Schematic views of the primary scalp pair, E9 (right) and E22 (left): (**a**) frontal view; (**b**) top view; (**c**) left-lateral view. Circles identify the selected electrodes. The secondary periocular pair, E14/E21, is evaluated separately [29]. Supplementary Section S1 lists nominal coordinates for both pairs from the standard HydroCel-128 montage [30].

We used the raw ZuCo 1.0 EEG and eye-tracking files from the three reading tasks. The primary scalp analysis uses E22 (left) and E9 (right), shown in Figure 2. The secondary analysis uses E21 and E14, which the ZuCo data descriptor classifies as periocular EOG sites. The two anatomical pairs are evaluated separately on the same eligible recording blocks [29].

The source study used a 128-channel HydroCel system sampled at 500 Hz and reported a Cz acquisition reference and contact impedances below 40 kOhm. The raw files contain 128 channels, with the reference field recorded as “common”. We retain the raw-file reference without additional re-referencing. The inputs are taken before the ocular-artefact processing used to generate the distributed 105-channel preprocessed EEG [29].

After restricting each recording to the common EEG and eye-tracker acquisition period, each input channel is filtered with a fourth-order Butterworth bandpass from 1 to 25 Hz and a 50 Hz notch with quality factor of 30. Both filters are applied forward and backward. Each channel is then centred and divided by its standard deviation over the entire retained block. No ICA, EOG regression, or ocular-component removal is applied before detection. This preprocessing is offline and uses block-level information.

#### 2.3.2. Synchronization, Eligibility, and Blink Reference

Reference blink intervals are the parser-defined events in the raw eye-tracker blink-event field. They are obtained independently of the EEG detector and have not been manually adjudicated. Shared EEG and eye-tracker trigger sequences define an affine clock mapping from eye-tracker timestamps to EEG sample indices, so alignment depends on acquisition triggers rather than blink waveforms.

The alignment requires at least five shared triggers, at least 90% of matched triggers within 20 ms of the fitted mapping, and a clock slope within 1% of the nominal sampling ratio. Intervals crossing the common acquisition boundary are omitted. At least 10 s of common data and finite, non-flat input channels are required. Among retained blocks, the largest 95th-percentile trigger residual was 4.88 ms and the largest individually retained residual was 19.51 ms. Supplementary Section S1 gives the synchronization procedure and file-level exclusions.

The available archive contains 273 raw EEG recording blocks. Three lack the paired eye-tracker file and ten fail the stated synchronization criteria, leaving 260 blocks from 12 participants, 1291.61 min of common recording, and 8611 reference blink intervals. We use all eligible blocks without a blink-rate or detection-performance cutoff. A block is a task recording file, not an independent participant. Task-specific counts are listed in Table 2.

### 2.4. Comparators and Event Scoring

The direct controls are a single-channel amplitude rule, thresholding of the bilateral average, a rule requiring peaks in both channels within 80 ms, and the proposed candidate pipeline with correlation as its only bilateral check. We also evaluate the official BLINKER implementation and a logistic-regression candidate classifier trained with leave-one-participant-out evaluation. Supplementary Section S2 reports exploratory two-channel ICA and synthetic-template controls, including the heuristic component selection used by the ICA implementation.

The amplitude rules use μ(|x|)+3σ(|x|), 50–700 ms intervals, a 0.3h boundary level, and a 200 ms merge gap. The correlation-only control retains the proposed candidate, duration, and slope rules and the 0.82 correlation cutoff, while disabling the other bilateral checks. BLINKER uses its official default quality criteria on the same two preprocessed input channels, selects the channel internally, and returns zero events when its quality checks reject both channels. This comparison applies the common preprocessing before BLINKER’s event extraction and quality checks; the adaptation is specified in Supplementary Section S2 [13]. The supervised control uses nine candidate features, training-only standardization, L2 logistic regression with C=1, balanced class weights, and a fixed probability cutoff of 0.5.

A detection is eligible to match a reference interval when its overlap with the reference expanded by 50 ms on each side is at least half the shorter unexpanded interval. We choose a one-to-one matching that first maximizes the number of matched pairs and then favours greater overlap. Each detection and each reference can contribute to at most one true positive. Unmatched detections and references are false positives and false negatives, respectively. We repeat the proposed-method scoring with 0, 25, and 100 ms tolerance as a sensitivity analysis.

For each participant, counts and exposure are summed across retained blocks before computing

(7) $$\begin{array}{cc} P & =\frac{TP}{TP+FP},\;R=\frac{TP}{TP+FN},\;F_{1}=\frac{2TP}{2TP+FP+FN}, \end{array}$$

(8) $$\begin{array}{cc} FP/\min & =\frac{FP}{\mathrm{retained}\;\mathrm{duration}\;\mathrm{in}\;\mathrm{minutes}}. \end{array}$$

Precision is set to zero if a method returns no detections. All participants have reference events. Reported means weight participants equally; event-pooled values are supplied separately. FP/min expresses the number of false events per minute of evaluated recording.

### 2.5. Threshold Selection, Ablations, and Perturbations

For each held-out participant, amplitude thresholds for the simple controls are selected using the other 11 participants. The search grid is 1.0 to 6.0 in steps of 0.5. We choose the threshold whose training-participant mean recall is closest to that of the frozen proposed detector; ties favour fewer false positives and then the larger threshold. The selected threshold is applied unchanged to every block of the held-out participant, and the achieved test recall is reported for each method. The complete predefined threshold sweep is supplied in Supplementary Materials S2.

Each ablation removes one verification group while leaving the others unchanged. Removing dominance disables both the median and quantile conditions. A candidates-only control removes all four bilateral checks but retains candidate generation, duration, slope, and merging. Each comparison measures the effect of the omitted check within the implemented pipeline.

Natural asymmetry is the mean absolute channel difference divided by the mean absolute channel sum. Within each channel pair, blocks are divided into low, middle, and high tertiles without using blink labels or detection outcomes. All three strata are reported. This signal-defined index describes bilateral imbalance within the evaluated recordings; its physiological or electrode-contact source is not measured.

Perturbations are added after filtering and standardization, with their amplitudes scaled by the standard deviation of the original symmetric component. The unilateral conditions add Gaussian noise of scale 0.3 or 0.6 and a slow random walk to the left channel. Additional conditions add either a shared Gaussian disturbance or independent Gaussian disturbances to both channels at scale 0.6. A transient-mixture condition adds brief unequal pulses and independent bursts at times drawn without reference to blink labels. These controlled perturbations test sensitivity to unilateral, bilateral, and transient signal disturbances.

Supplementary Section S3 specifies the distributions, random seed, and full generation equations. Perturbed signals are not filtered or standardized again. All methods within a comparison receive the same disturbed signals and reference intervals.

### 2.6. Statistical Analysis and Computational Cost

The participant is the unit of statistical analysis. We report participant means, standard deviations, and 95% percentile bootstrap confidence intervals from 20,000 resamples of the 12 participants. Task and stratum summaries resample the participants represented in that group. Principal comparisons use two-sided exact paired sign-flip tests over all 4096 sign assignments. Holm correction is applied jointly to 12 tests: F1 and FP/min for the proposed detector versus six principal comparators on the scalp pair. Paired mean differences, their bootstrap intervals, standardized paired effects, and exact and adjusted *p*-values are reported in Supplementary Table S3. Other analyses are descriptive unless a test family is explicitly specified.

For *N* samples, *K* candidates, and maximum window length *W*, signal scans and event checks require *O*(*N* + *KW*) work, with up to *O*(*K* log *K*) work for peak ordering and merging. Quantile calculations use selection operations in the reference implementation. The offline arrays require *O*(*N* + *K*) storage. We benchmark the Python 3.12.14 detectors on one block per participant selected by file order, after one warm-up and with seven repeated runs. Timing excludes file loading, scoring, and plotting; preprocessing is measured separately. The memory measure is the peak allocation tracked by Python during detection, excluding preallocated inputs and untracked native allocations.

## 3. Results

### 3.1. Overall Performance and Participant Variability

On the scalp pair, the proposed detector achieved a participant-mean precision of 0.731, recall of 0.774, F1 of 0.734, and 1.469 false positives per minute. The corresponding 95% confidence intervals were 0.639–0.822, 0.675–0.861, 0.661–0.805, and 0.934–2.126. Table 3 reports the fixed-setting comparison. The periocular pair yielded precision of 0.728, recall of 0.842, F1 of 0.768, and 1.605 false positives per minute; full pair-specific results are given in Supplementary Table S2.

At fixed settings, the proposed detector produced fewer false positives than the single-channel rule and bilateral-average rule, with paired differences of −2.444 and −1.945 FP/min, respectively; both Holm-adjusted *p*-values were 0.005859. The corresponding F1 differences were not significant after correction. Relative to correlation alone, the full detector reduced FP/min by 0.152 (adjusted *p* = 0.009766), with a mean recall of 0.774 compared with 0.825. Its F1 was similar to the both-channel rule and the supervised control. The verification stage therefore shifts the operating point towards fewer false positives, with a corresponding change in sensitivity.

BLINKER’s default quality checks rejected both input channels in 126 of the 260 scalp blocks. Those blocks contribute zero detections to the common-cohort evaluation. Its reported recall therefore includes the effect of quality rejection under the specified preprocessing. Participant-specific results and block status counts accompany the result tables.

### 3.2. Controlled Perturbations

**Figure 3:**
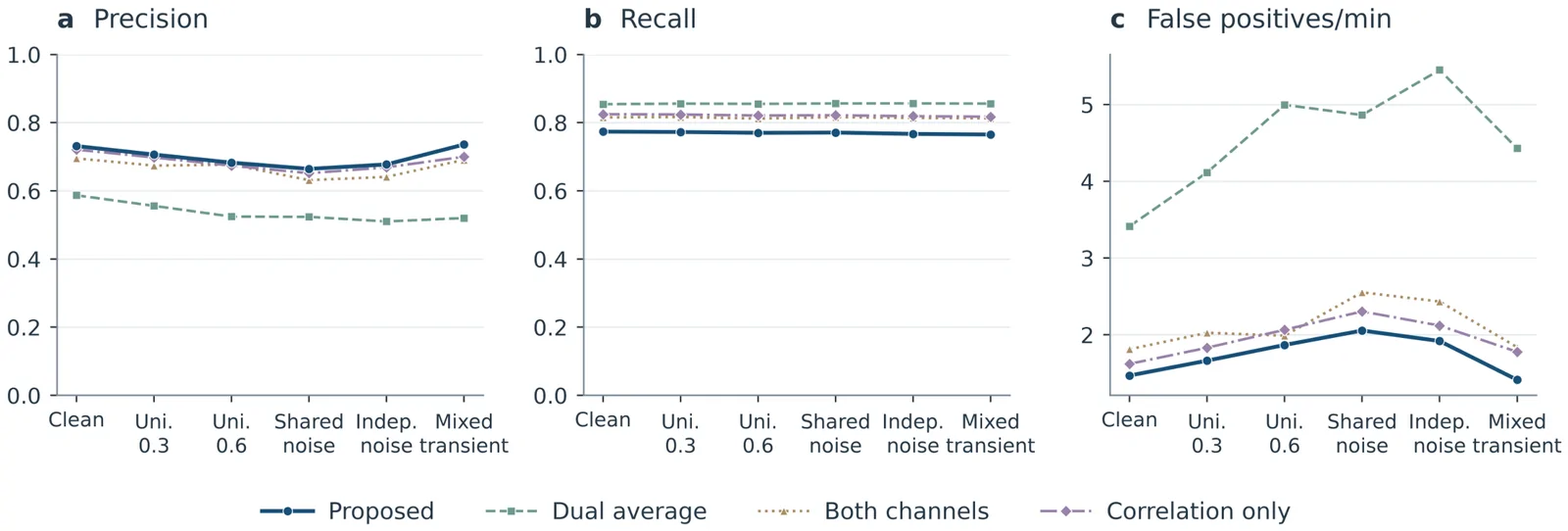
Scalp-pair performance under controlled perturbations. Curves show means across participants at fixed detector settings. Uni.: unilateral Gaussian noise plus drift; Shared: shared bilateral Gaussian noise; Indep.: independent bilateral Gaussian noise. Complete numerical results and uncertainty are supplied in the Supplementary Tables.

Across all five perturbation conditions, the proposed detector produced fewer false positives than the single-channel, bilateral-average, both-channel, and correlation-only controls at their fixed settings. Its recall remained between 0.765 and 0.772, compared with 0.774 on the unperturbed recordings. Scalp FP/min increased from 1.469 to 1.663 and 1.868 under unilateral scales of 0.3 and 0.6, to 2.056 under shared bilateral noise, and to 1.919 under independent bilateral noise. All four Gaussian-noise increases were significant within the ten-test perturbation family (Holm-adjusted *p* = 0.004883 each). The transient mixture reduced recall by 0.0085 (adjusted *p* = 0.046875). Figure 3 and Supplementary Section S3 report the comparisons and complete numerical results.

### 3.3. Natural Asymmetry and Component Ablations

**Figure 4:**
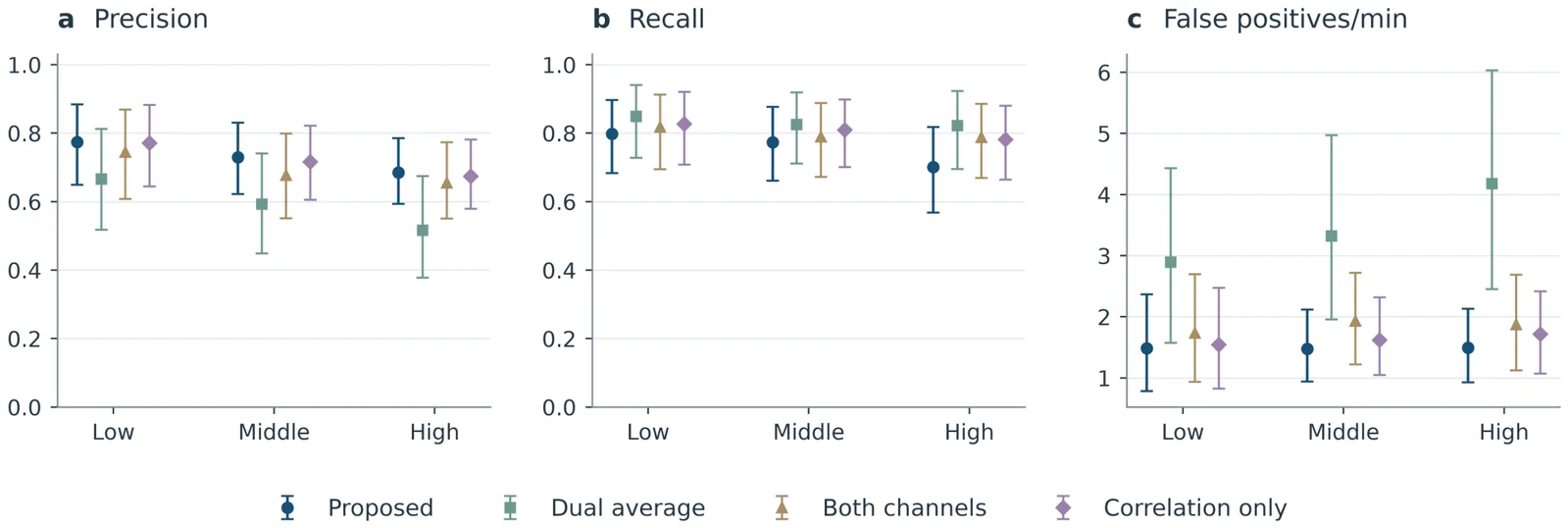
All natural-asymmetry strata for the scalp pair. Points are participant means within each stratum; error bars are participant-bootstrap 95% intervals. Blocks contribute to one stratum, while a participant can contribute to more than one. These are descriptive within-dataset comparisons.

The proposed scalp detector maintained similar FP/min across low, middle, and high natural-asymmetry strata (Figure 4), with means of 1.484, 1.475, and 1.494. Mean precision was 0.774, 0.729, and 0.685, and recall was 0.798, 0.773, and 0.701, respectively. Thus, higher asymmetry was associated with lower sensitivity and little change in false-positive rate. The strata contained blocks from 9, 10, and 10 participants, so these descriptive comparisons also reflect differences in participant composition.

The ablations identify how individual checks shape the detection decision (Table 4). Including amplitude symmetry reduced scalp FP/min from 1.623 to 1.469, while recall decreased from 0.827 to 0.774 and F1 from 0.758 to 0.734. Correlation also contributed to false-positive rejection. Removing dominance left the final detections unchanged in the clean fixed-setting analysis, showing overlap with the remaining criteria under those conditions. Removing the timing check caused small changes after merging. These results identify amplitude symmetry as a direct control of the sensitivity and false-positive trade-off in the implemented rule.

Across merge gaps of 100–300 ms, scalp F1 ranged from 0.731 to 0.735 and recall from 0.768 to 0.780. The 200 ms development setting is retained throughout the main analysis. Changing matching tolerance from 0 to 100 ms changed F1 from 0.728 to 0.740. Full sensitivity values are given in Supplementary Table S5.

### 3.4. Fixed Settings and Threshold Selection

**Figure 5:**
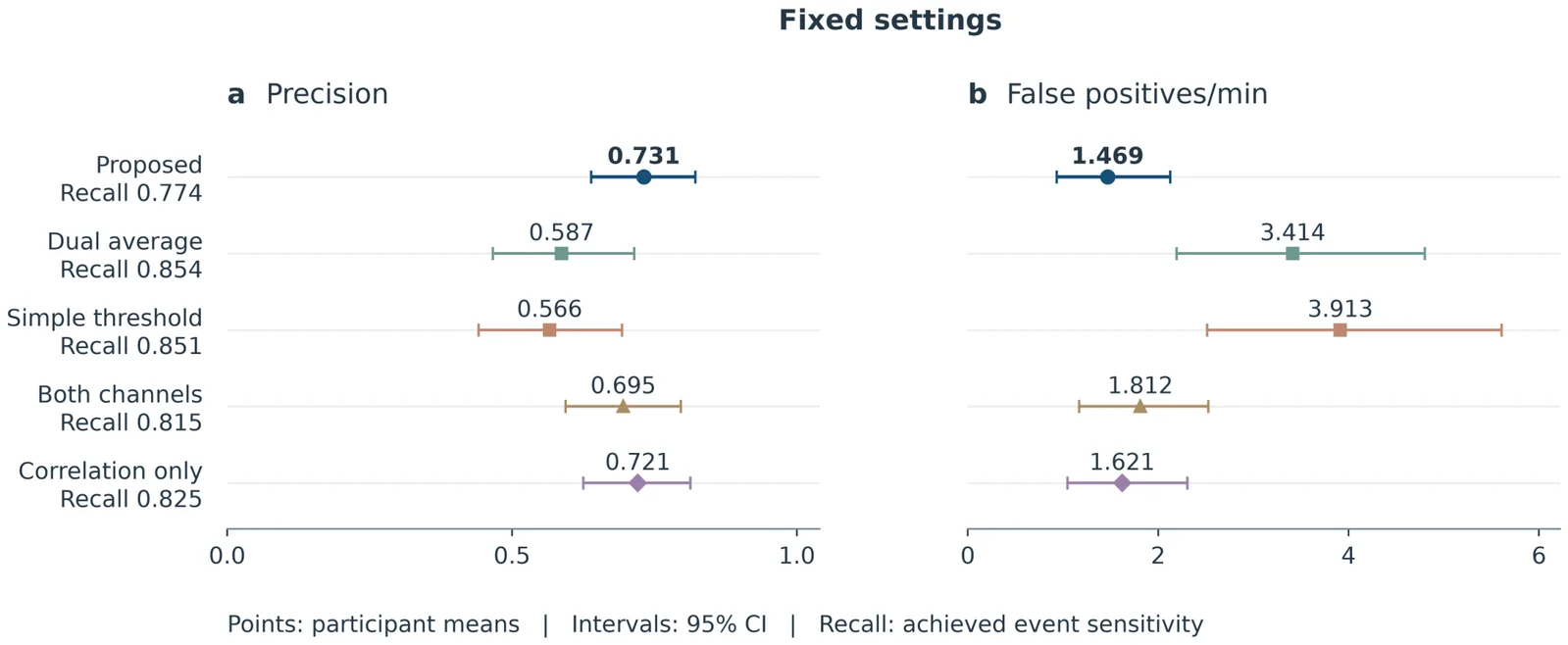
Fixed-setting scalp-pair screening performance. Points show participant means with 95% participant-bootstrap confidence intervals (12 participants). Recall is listed beside each method. Panel (**a**) shows precision and panel (**b**) shows false positives per minute. All five rule-based methods use their stated fixed settings. The participant-held-out threshold comparison is reported separately in Supplementary Table S4 and Figure S1.

Figure 5 shows fixed-setting screening performance. Among the five rule-based methods, the proposed detector achieved the lowest mean FP/min (1.469), with precision 0.731 and recall 0.774. Participant-held-out control threshold selection quantified the effect of operating-point choice. Several simple controls reached lower FP/min at similar recall; the complete comparison is reported in Supplementary Table S4 and Figure S1. Supplementary Materials S2 provide the full threshold sweep and each held-out choice.

### 3.5. Runtime and Memory

On an Apple M1 Pro computer with 16 GB memory, the proposed Python 3.12.14 detector required a median of 2.13 ms per minute of recorded scalp data across the 12 benchmark blocks. The corresponding values were 0.34 ms for the single-channel rule, 0.38 ms for bilateral averaging, and 1.44 ms for correlation alone. The proposed detector’s median tracked peak allocation was 4.32 MiB. Table 5 reports the common implementation comparison. These measurements establish a low computational cost for the offline detection stage.

## 4. Discussion

### 4.1. What Bilateral Verification Adds

Bilateral verification makes candidate rejection traceable to waveform correlation, timing, relative amplitude, and symmetric-component dominance. These measurements describe related aspects of the same event. For identical waveforms differing only by a positive scale factor *r* no greater than one, amplitude asymmetry is 1 − *r* and the antisymmetric-to-symmetric ratio is (1 − *r*)/(1 + *r*). This relationship explains the overlapping effect of amplitude and dominance checks in the clean-data ablation. Noise and temporal shifts modify that relationship, allowing the checks to characterize different forms of bilateral mismatch.

**Figure 6:**
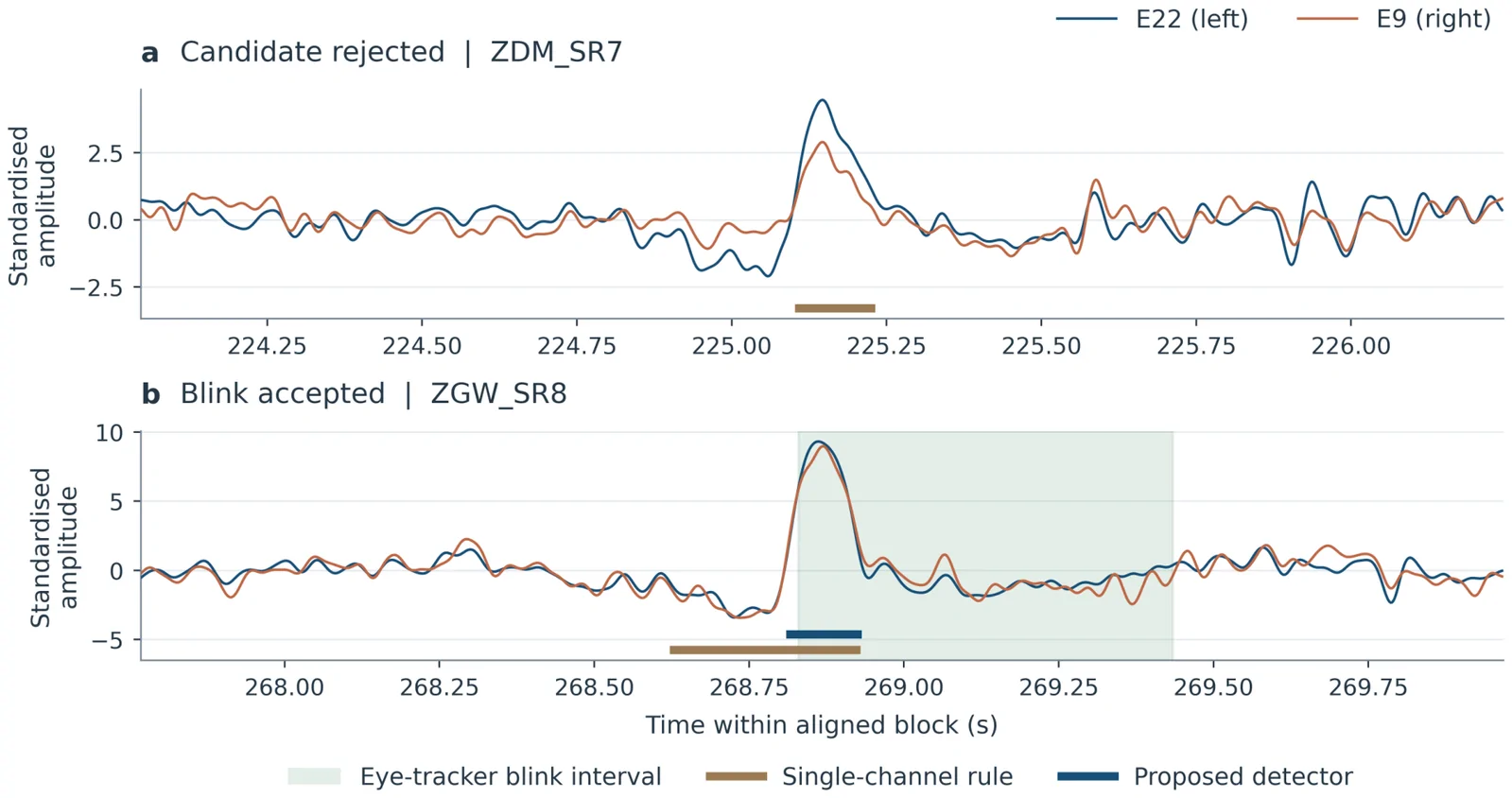
Examples of scalp-pair detection outcomes. Green shading marks the eye-tracker reference interval. Lower bars show the single-channel rule in brown and the proposed detector in blue. Panel (**a**) shows a candidate rejected by bilateral verification; panel (**b**) shows a reference interval matched by the proposed detector but unmatched by the single-channel rule under the stated event-matching criterion.

Figure 6 illustrates a rejected candidate and an accepted reference blink on the scalp pair. The examples were selected by detection outcome to show the event-level decisions.

The bilateral criteria also define identifiable failure modes. A true blink that overlaps a horizontal saccade or has unequal bilateral amplitudes can be rejected. Conversely, vertical eye movements, brow movement, or bilateral frontalis activity may satisfy the checks. Absolute-amplitude candidate generation accommodates either signal polarity, including non-blink transients. Blink identification therefore depends on the combined waveform and bilateral criteria rather than a universal polarity rule.

### 4.2. Consequences of Missed and Spurious Detections

Missed blinks leave ocular contamination in data passed to a downstream decoder and may bias spectral or event-related features. False positives can remove useful EEG, reduce the number of usable trials, or distort an analysis when rejected segments are concentrated in one condition. The relative costs depend on the decoder and on whether detections trigger censoring, correction, or a quality warning. Precision and recall jointly describe the reliability and completeness of positive predictions, which is especially useful in imbalanced classification [31]. Reporting recall together with false positives per minute makes this trade-off explicit when choosing a detector for a downstream analysis.

Blink detection can guide subsequent artefact correction. Single-channel correction methods include singular spectrum analysis combined with continuous wavelet analysis and k-means clustering [32], as well as adaptive spectrum-template matching [33]. AFFiNE fits a blink-related reference signal before adaptive filtering and evaluates preservation of the underlying P300 response [34]. The proposed detector supplies event intervals that can support a separate correction or rejection stage while keeping the detection decision open to inspection.

### 4.3. Contact Quality and Deployment

ZuCo’s acquisition protocol required contact impedances below 40 kOhm. Our detector evaluates the recorded waveforms and does not receive impedance measurements. Severe cap displacement can change the relationship between the selected anatomical sites and invalidate bilateral comparisons. Persistent placement or contact problems therefore call for an acquisition-quality check and electrode inspection or repositioning. Signal asymmetry alone does not identify the physical cause of poor recording quality.

The benchmark uses recordings from a seated laboratory cap and an offline implementation. Forward-backward filtering, block-wise normalization, and the boundary search use future samples. Streaming deployment would require causal filters, running statistics, and a specified decision delay. The measured computational cost provides an implementation baseline; device latency, memory, and energy use require measurements on the target hardware.

### 4.4. Scope and Limitations

The evaluation spans 12 participants and three reading tasks acquired with the same hardware. It characterizes task variation within ZuCo. Transfer of the fixed detector settings to independent recordings and wearable montages remains to be tested.

## 5. Conclusions

Physiology-informed bilateral verification makes blink-event decisions explicit using two frontal channels. Evaluation on 260 eligible ZuCo blocks shows reduced false-positive rates relative to single-channel thresholding and bilateral averaging at fixed settings, while component ablations quantify the error trade-offs introduced by the verification criteria. The offline implementation required a median of 2.13 ms per minute of recorded data. The method provides an interpretable and computationally inexpensive approach to blink-event screening in frontal EEG.

## Figures and Tables

**Table 1:** Parameters of the frozen reference detector. All times are converted to samples by integer truncation.

Operation	Setting
Candidate amplitude	$\max\{\mu (|x_{S}|)+3.8\sigma (|x_{S}|),Q_{0.95}\left(\right|x_{S}\left|)\right\}$
Peak spacing/boundary level	12 samples at 500 Hz/0.3h
Boundary search/accepted duration	±350 ms/50–700 ms
Correlation smoothing/cutoff	20 ms moving average/0.82
Maximum peak-time difference	80 ms
Maximum amplitude asymmetry	0.32; channel amplitudes use 95th percentiles
Symmetric dominance	Median ratio ≥1.3 and quantile ratio ≤0.7
Minimum slope	$1.5\sigma (\Delta x_{S})$
Merge gap	<200 ms, end to next onset
Disabled options	Adaptive correlation and additional shape checks

**Table 2:** Eligible ZuCo recording blocks. SR: sentiment reading; NR: normal reading; TSR: task-specific reading. Participants overlap across tasks.

Task	Participants	Blocks	Minutes	Blinks
SR	12	90	456.41	3294
NR	12	71	500.70	2808
TSR	11	99	334.50	2509
Total	12	260	1291.61	8611

**Table 3:** Scalp-pair performance at fixed settings, summarized as mean ± SD across 12 participants. Counts are pooled within each participant first. Logistic LOSO denotes the participant-held-out supervised control; BLINKER includes quality-rejected blocks.

Detector	Precision	Recall	F1	FP/min
Proposed	0.731 ± 0.169	0.774 ± 0.172	0.734 ± 0.133	1.469 ± 1.117
Simple threshold	0.566 ± 0.234	0.851 ± 0.158	0.654 ± 0.198	3.913 ± 2.878
Dual average	0.587 ± 0.232	0.854 ± 0.162	0.674 ± 0.196	3.414 ± 2.433
Both channels	0.695 ± 0.188	0.815 ± 0.167	0.738 ± 0.158	1.812 ± 1.268
Correlation only	0.721 ± 0.175	0.825 ± 0.153	0.756 ± 0.142	1.621 ± 1.174
BLINKER	0.683 ± 0.377	0.450 ± 0.346	0.478 ± 0.341	0.968 ± 2.380
Logistic LOSO	0.649 ± 0.212	0.854 ± 0.152	0.722 ± 0.176	2.593 ± 2.120

**Table 4:** Scalp-pair component ablations, participant means. Other settings are held fixed.

Variant	Precision	Recall	F1	FP/min
Proposed	0.731	0.774	0.734	1.469
Without correlation	0.715	0.794	0.735	1.626
Without timing	0.731	0.771	0.732	1.467
Without amplitude symmetry	0.721	0.827	0.758	1.623
Without dominance	0.731	0.774	0.734	1.469
Correlation only	0.721	0.825	0.756	1.621
Candidates only	0.706	0.855	0.760	1.803

**Table 5:** Offline Python detector cost on Apple M1 Pro, macOS 15.7.3, Python 3.12.14. Each block contributes its median of seven runs. IQR is across the 12 blocks. Memory excludes preallocated inputs and untracked native allocations.

Detector	ms/Recorded min	IQR	Tracked MiB
Proposed	2.13	1.82–2.46	4.32
Simple threshold	0.34	0.32–0.36	2.70
Dual average	0.38	0.36–0.40	4.86
Both channels	0.69	0.64–0.73	2.71
Correlation only	1.44	1.31–1.61	4.32
ICA two-channel	4.75	4.49–5.23	10.81
Template	8.72	6.91–16.30	5.47

## Data Availability

The raw data are available in ZuCo 1.0 at https://doi.org/10.17605/OSF.IO/Q3ZWS. Supplementary Materials S1 and S2 provide the analysis specifications, code, parameter files, input-file manifest, and derived results for this reanalysis. Raw participant recordings are not duplicated in the supplementary package.

## Supplementary Materials

The following supporting information can be downloaded at https://www.mdpi.com/article/10.3390/bioengineering13101208/s1. Supplementary Materials S1: Supplementary methods and results. Table S1: Excluded raw recording blocks. Table S2: Complete-cohort F1 and FP/min, participant means with 95% bootstrap confidence intervals. All four metrics and SDs are supplied in summary.csv. Table S3: Principal paired comparisons on the scalp pair. Negative FP/min differences favour the proposed detector. Table S4: Achieved participant-held-out operating points. Table S5: Merge-gap and matching-tolerance sensitivity, scalp pair. A change affects only the named setting. Figure S1: Participant-held-out control-threshold comparison for the scalp pair. Points show participant means with 95% participant-bootstrap confidence intervals (12 participants). Recall is listed beside each method. Panel (a) shows precision and panel (b) shows false positives per minute. Each control’s amplitude threshold is selected using the other 11 participants and applied unchanged to the held-out participant. The proposed detector retains its frozen settings. Supplementary Materials S2: Reproducibility code, pinned dependencies, frozen detector parameters, input-file manifest with hashes, derived results, and figure provenance.

## Abbreviations

The following abbreviations are used in this manuscript:

EEG	Electroencephalography
EOG	Electrooculography
ICA	Independent component analysis
