# Supplementary material for: Physiology-Informed Blink Artefact Detection from Bilateral Frontal EEG Under Asymmetric Interference
Source: Bioengineering (Basel). 2026 Oct 16;13(10):1208. doi: 10.3390/bioengineering13101208 (PMC13701908; doi:10.3390/bioengineering13101208)
Supplement: Supplementary file 1 [file bioengineering-13-01208-s001.zip › bioengineering-4557656-Supplementary-Materials-S1.pdf]

Supplementary Materials S1

Physiology-Informed Blink Artefact Detection from Bilateral Frontal EEG Under Asymmetric Interference

Bioengineering manuscript bioengineering-4557656

S1. Cohort, provenance, and synchronization

The inventory covers all 273 raw EEG blocks present in the available ZuCo 1.0 archive. It contains 12 participants, with all three task types represented. File names using SNR for the later sentiment-reading blocks are paired with the corresponding SR eye-tracker file. Three paired eye-tracker files are absent. Ten further blocks fail the fixed synchronization checks. Table S1 lists every exclusion. Eligibility is determined before detector evaluation, without a blink-rate, amplitude, or performance cutoff.

EEG event latencies are converted from one-based samples to zero-based samples. Trigger types are parsed as integers. The EEG and eye-tracker trigger-code sequences are matched in order with SequenceMatcher (autojunk disabled); matched timestamps are fitted with an affine mapping. Up to four iterations discard trigger residuals greater than 20 ms. At least five triggers and 90% inliers are required, with fitted slope within 1% of the nominal 0.5 samples/ms. The common recording period is the intersection of EEG and mapped eye-tracker timestamps. Reference blink onsets and offsets are rounded to samples. Duplicate intervals are removed, and intervals not wholly inside the common period are excluded. Source file hashes, common-period indices, alignment residuals, and included reference counts are recorded in the supplied manifest.

The raw-file channel labels identify the inputs directly. Nominal coordinates in cm are E22 (-2.695, 8.885, 1.088), E9 (2.695, 8.885, 1.088), E21 (-1.270, 9.479, -0.947), and E14 (1.270, 9.479, -0.947). The standard montage coordinates are not individual digitizations. The acquisition reference reported in the ZuCo descriptor is Cz; the retained raw metadata field says “common”. This reanalysis retains the raw reference and performs no additional re-referencing or ocular-component removal.

The revised evaluation replaces the earlier 46-block comparison, whose selection involved blink rate and detection performance. The old Table 1 FP column represented mean false-positive counts per block, although it was labeled FP/min; the old template F1 standard deviation was also transcribed incorrectly (0.015 instead of approximately 0.148). The reanalysis recomputes all counts, rates, uncertainty, figures, and tables using one synchronized cohort and one-to-one event matching. The revised values are not a rescaling of the earlier selected-cohort results. The supplied reference code also contains development settings different from the limited parameters archived with the earlier comparison. The complete parameter file used here is frozen and included in Supplementary Materials S2.

Table S1: Excluded raw recording blocks.

Block	Reason
ZDN_SR6	shared-trigger residual exceeds 20 ms
ZDN_SR7	paired ET file is absent
ZDN_SR8	paired ET file is absent
ZKW_SR6	paired ET file is absent
ZKB_TSR1	shared-trigger residual exceeds 20 ms
ZKB_TSR2	shared-trigger residual exceeds 20 ms
ZKB_TSR3	shared-trigger residual exceeds 20 ms
ZKB_TSR4	shared-trigger residual exceeds 20 ms
ZKB_TSR5	shared-trigger residual exceeds 20 ms
ZKB_TSR6	shared-trigger residual exceeds 20 ms
ZKB_TSR7	shared-trigger residual exceeds 20 ms
ZKB_TSR8	shared-trigger residual exceeds 20 ms
ZKB_TSR9	shared-trigger residual exceeds 20 ms

S2. Comparator implementations and complete results

The proposed detector uses the source implementation and the complete parameter file accompanying this revision. The main preprocessing is applied to the cropped raw pair, with no preliminary artifact removal. The order is fourth-order 1–25 Hz Butterworth filtering, 50 Hz notch filtering (quality factor 30), then channel-wise centering and standardization. Both filters use SciPy filtfilt. For the correlation and amplitude-symmetry calculations only, a 10-sample moving average is applied inside each candidate window using convolution with same-length zero padding. Dominance and peak lag use the unsmoothed preprocessed signals. The slope check is active; adaptive correlation and the additional half-width, rise/fall, and area checks are disabled.

The single-channel control uses the left channel. The bilateral-average control uses $(L + R)/2$ without bilateral verification. Their default amplitude multiplier is 3, with 50–700 ms duration, 24 ms candidate spacing at 500 Hz, $0.3h$ boundary level, and 200 ms merging. The both-channel control runs the same single-channel detector separately on each channel and retains a left-channel event if a right-channel peak occurs within 80 ms. The correlation-only control uses the proposed candidate pipeline and 0.82 correlation cutoff, but disables amplitude symmetry, timing, and dominance.

The two-channel ICA control applies scikit-learn FastICA (two components, unit-variance whitening, random state 42, maximum 200 iterations). The component is selected by the product of mixing-weight symmetry and component variance, followed by amplitude thresholding with multiplier 2.5. No trained ICLabel classifier is used. This is an exploratory two-channel control, not a substitute for high-density ICA. The template control uses the supplied synthetic 200 ms negative Gaussian waveform and a correlation cutoff of 0.6. These simple controls are described by their actual implementations rather than presented as exact reproductions of the older papers cited for general context.

BLINKER is the official EEG-Blinks implementation at commit `50fb6212897d98273ca7b5882c69af7a54ba68fc`. The wrapper calls extractBlinks and extractBlinkProperties on the two common preprocessed channels. Default morphology and quality parameters are retained: minGoodBlinks 10, standard-deviation threshold 1.5, blink-amplitude range 3–50, good-blink ratio 0.7, top/bottom fit thresholds 0.98/0.90, and z-threshold pairs (0.90,2) and (0.98,5). Event boundaries are the accepted fits' leftZero and rightZero fields. Blocks rejected by the quality checks contribute zero detections and all reference events remain eligible to be missed. There were 137 such periocular blocks and 126 scalp blocks; no wrapper execution errors occurred. This adaptation standardizes preprocessing and does not reproduce BLINKER's complete native filtering workflow.

The supervised control uses candidates with amplitude threshold $\max(\mu(|S|) + \sigma(|S|), Q_{0.95}(|S|))$, the same boundary search, and 50–700 ms duration. Its nine features are absolute peak, duration, smoothed correlation, absolute peak lag, amplitude asymmetry, log-transformed median dominance,

antisymmetric/symmetric 95th-percentile ratio, normalized maximum slope, and signed symmetric peak. Candidate labels are based only on the training participants' reference overlap. StandardScaler and L2 logistic regression ($C = 1$, balanced class weights, maximum 2000 iterations) are fitted to the other 11 participants. A fixed probability threshold of 0.5 is used, and accepted intervals are merged at 200 ms. Every held-out participant is excluded from feature scaling and model fitting. This control does not establish performance relative to all supervised architectures.

Table S2: Complete-cohort F1 and FP/min, participant means with 95% bootstrap confidence intervals. All four metrics and SDs are supplied in summary.csv.

Pair	Detector	F1 [95% CI]	FP/min [95% CI]
scalp	Proposed	0.734 [0.661, 0.805]	1.469 [0.934, 2.126]
scalp	Simple threshold	0.654 [0.545, 0.758]	3.913 [2.514, 5.606]
scalp	Dual average	0.674 [0.570, 0.781]	3.414 [2.195, 4.802]
scalp	Both channels	0.738 [0.650, 0.821]	1.812 [1.169, 2.528]
scalp	Correlation only	0.756 [0.674, 0.830]	1.621 [1.049, 2.306]
scalp	BLINKER	0.478 [0.289, 0.658]	0.968 [0.137, 2.416]
scalp	Logistic LOSO	0.722 [0.622, 0.812]	2.593 [1.578, 3.854]
scalp	ICA two-channel	0.599 [0.479, 0.725]	5.670 [3.302, 8.649]
scalp	Template	0.412 [0.280, 0.557]	19.464 [11.094, 29.875]
periocular	Proposed	0.768 [0.683, 0.847]	1.605 [0.986, 2.335]
periocular	Simple threshold	0.703 [0.604, 0.797]	2.900 [1.912, 4.032]
periocular	Dual average	0.695 [0.598, 0.792]	2.999 [1.995, 4.048]
periocular	Both channels	0.721 [0.627, 0.813]	2.289 [1.481, 3.155]
periocular	Correlation only	0.775 [0.691, 0.854]	1.639 [1.029, 2.373]
periocular	BLINKER	0.513 [0.347, 0.682]	0.781 [0.178, 1.733]
periocular	Logistic LOSO	0.741 [0.650, 0.830]	2.129 [1.291, 3.117]
periocular	ICA two-channel	0.622 [0.513, 0.733]	4.748 [3.045, 6.833]
periocular	Template	0.426 [0.302, 0.562]	16.555 [10.340, 24.437]

S3. Statistical comparisons and perturbation specification

Principal tests compare 12 paired participant values on the scalp pair. The exact two-sided sign-flip p-value is the fraction of all 2^{12} sign assignments whose absolute mean difference is at least the observed value. Holm adjustment covers six comparators and two outcomes jointly. Differences are proposed minus comparator; d_z is the mean paired difference divided by its sample standard deviation. Bootstrap confidence intervals resample participants as paired units 20,000 times with seed 20261006. Intervals are descriptive percentile intervals, not multiplicity-adjusted intervals, and need not invert the exact permutation test. Participant dependence within a task or across blocks is not treated as extra replication.

Table S3: Principal paired comparisons on the scalp pair. Negative FP/min differences favor the proposed detector.

Comparator	Metric	Δ	95% CI	d_z	Exact p	Holm p
Simple threshold	F1	0.080	[0.008, 0.150]	0.609	0.060059	0.360352
Simple threshold	FPmin	-2.444	[-3.887, -1.320]	-1.020	0.000488	0.005859
Dual average	F1	0.060	[-0.017, 0.127]	0.447	0.159180	0.795898
Dual average	FPmin	-1.945	[-3.083, -1.064]	-1.038	0.000488	0.005859
Both channels	F1	-0.004	[-0.059, 0.034]	-0.050	0.936523	1.000000
Both channels	FPmin	-0.343	[-0.498, -0.190]	-1.205	0.002441	0.019531
Correlation only	F1	-0.022	[-0.062, 0.004]	-0.341	0.387207	1.000000
Correlation only	FPmin	-0.152	[-0.223, -0.084]	-1.176	0.000977	0.009766
BLINKER	F1	0.256	[0.106, 0.418]	0.880	0.004883	0.034180
BLINKER	FPmin	0.502	[-0.471, 1.173]	0.322	0.307617	1.000000
Logistic LOSO	F1	0.012	[-0.052, 0.070]	0.108	0.725586	1.000000
Logistic LOSO	FPmin	-1.124	[-1.796, -0.542]	-0.976	0.000977	0.009766

Let $s = \mathrm{SD}(S)$ before disturbance and z_t denote independent standard Gaussian samples. The unilateral disturbance is $L'_t = L_t + qsz_t + d_t$, $R'_t = R_t$, for $q = 0.3$ or 0.6, with $d_t = \sum_{j \le t} \epsilon_j$ and $\epsilon_j \sim N(0, (0.1s/f_s)^2)$. Shared bilateral noise uses the same $0.6sz_t$ in both channels; independent bilateral noise uses separate samples. For the transient mixture, the number of pulse times is $\max(1, \lfloor 2T_{\min} \rfloor)$, where $T_{\min}$ denotes the retained recording duration in minutes. Pulse times are drawn uniformly between one second and one second before the end. Each pulse is evaluated from -0.2 to 0.8 s relative to its onset, with $p(t) = 5s\exp(-\max(t, 0)/0.12)\mathbf{1}_{t\ge0}$. The left channel receives $p(t)$ and the right $0.4p(t)$; both also receive independent Gaussian bursts scaled by $1.5s\exp(-t^2/0.01)$. The seed is 20261006 plus the sum of the block identifier's byte values plus the left-channel cache index. The script specifies the draw order. No reference labels determine disturbance timing. Filtering and standardization are not repeated after injection.

The ten perturbation tests comprise changes from clean data in the proposed detector's scalp recall and FP/min for five conditions, corrected as one Holm family. They are separate from the principal comparator family. Full results are in noise_tests.csv and noise_summary.csv. These tests describe responses to the specified synthetic signals, not clinical or physical artifact categories.

S4. Held-out thresholds and sensitivity analyses

The test participant does not contribute to amplitude-threshold selection. Training recall is the equal-weight mean of the other participants' count-pooled recalls. The closest baseline training recall to the proposed detector's fixed-setting recall determines the threshold; ties use lower FP/min then larger multiplier. Achieved test recall can differ. The proposed detector remains at its frozen multiplier of 3.8, also included in its descriptive threshold curve. Full fold choices appear in loso_thresholds.csv. Because the original detector was developed on ZuCo, this experiment isolates threshold-selection leakage rather than providing an independent external test of the entire development process.

Table S4: Achieved participant-held-out operating points.

Pair	Method	Precision	Recall	F1	FP/min
scalp	Proposed	0.731	0.774	0.734	1.469
scalp	Simple threshold	0.840	0.760	0.770	0.775
scalp	Dual average	0.867	0.781	0.799	0.636
scalp	Both channels	0.795	0.790	0.751	1.623
scalp	Correlation only	0.855	0.766	0.783	0.695
periocular	Proposed	0.728	0.842	0.768	1.605
periocular	Simple threshold	0.625	0.845	0.678	5.906
periocular	Dual average	0.396	0.828	0.465	12.053
periocular	Both channels	0.657	0.837	0.721	2.289
periocular	Correlation only	0.579	0.841	0.623	7.687

The scalp-pair threshold-selected comparison is displayed in Figure S1 with its achieved recall and uncertainty. At similar recall, several threshold-selected simple controls reached lower FP/min than the frozen proposed detector. Table S4 retains all four metrics for both anatomical pairs, and Supplementary Materials S2 provide the full threshold sweep and each fold's selected value.

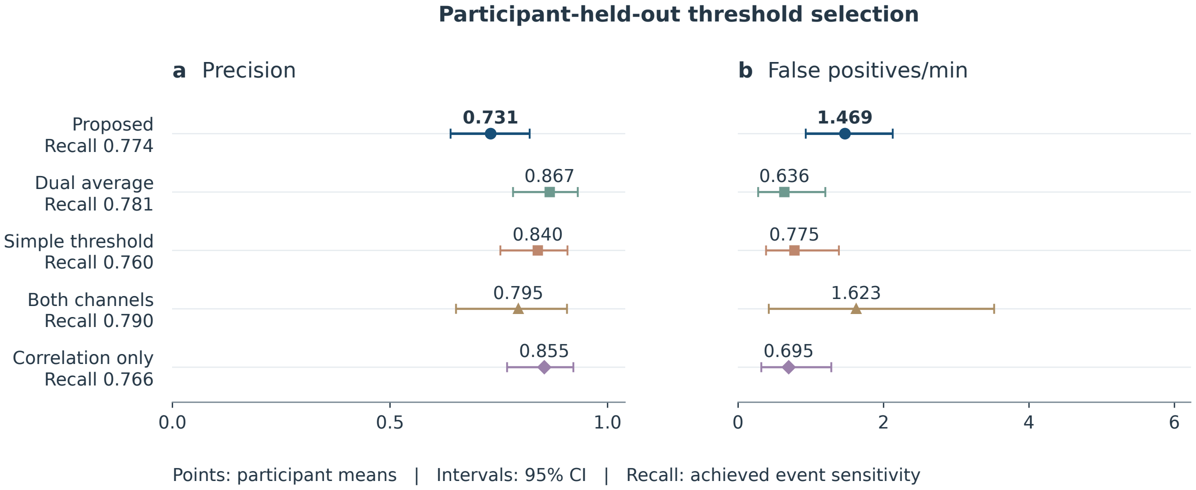

Figure S1: Participant-held-out control-threshold comparison for the scalp pair. Points show participant means with 95% participant-bootstrap confidence intervals (12 participants). Recall is listed beside each method. Panel (a) shows precision and panel (b) shows false positives per minute. Each control's amplitude threshold is selected using the other 11 participants and applied unchanged to the held-out participant. The proposed detector retains its frozen settings.

Table S5: Merge-gap and matching-tolerance sensitivity, scalp pair. A change affects only the named setting.

Setting	Precision	Recall	F1	FP/min
Merge 100 ms	0.721	0.780	0.731	1.569
Merge 150 ms	0.729	0.777	0.734	1.496
Default: 200 ms gap, 50 ms tolerance	0.731	0.774	0.734	1.469
Merge 250 ms	0.734	0.771	0.735	1.442
Merge 300 ms	0.736	0.768	0.734	1.423
Tolerance 0 ms	0.725	0.767	0.728	1.511
Tolerance 25 ms	0.729	0.772	0.732	1.480
Tolerance 100 ms	0.737	0.779	0.740	1.440

S5. Runtime, memory, and reproducibility files

Timing was measured on macOS 15.7.3, Apple M1 Pro, 16 GB memory, Python 3.12.14, with numeric-library thread limits set to one. The first included file for each participant was chosen before examining its detection performance. Each detector was warmed up once and measured seven times on the same preprocessed scalp block. Medians within blocks and then across the 12 blocks are reported. Preprocessing and detector timings exclude I/O; preprocessing took approximately 11 ms per benchmark block at the cross-block median. These are measurements of the supplied Python implementations, not algorithm-independent speed rankings. BLINKER runs in MATLAB and is not included in this common Python timing table. The runtime files retain its execution times for provenance only.

Peak allocation is measured in a separate detector run using tracemalloc. It excludes preallocated input arrays, interpreter startup, and native allocations not tracked by Python. It should not be read as total RAM or an embedded-memory requirement. No device power or energy measurement was made.

Supplementary Materials S2 contains code, pinned dependency versions, the frozen parameter file, a relative-path input manifest with hashes, block-level and participant-level metrics, threshold choices, statistical outputs, and figure provenance. The README describes reproduction. The original raw recordings remain available from ZuCo; they are not redistributed here. The published BLINKER repository and its GPL license are identified separately. All manuscript numbers are generated from the accompanying result tables.
